# Supplementary material for: A compact design of MIMO patch antenna with high gain and symmetrical radiation pattern
Source: PLoS One. 2025 Jul 10;20(7):e0325282. doi: 10.1371/journal.pone.0325282 (PMC12244613; doi:10.1371/journal.pone.0325282)
Supplement: Attachment — Submitted filename: Response to Reviewers.docx [file pone.0325282.s001.docx]

**Submission ID:** PONE-D-25-15830

**Original Article Title:** “A Compact Design of MIMO Patch Antenna with High Gain and Symmetrical Radiation Pattern”

**To:** Reviewer

**Re:** Response to reviewer

Dear Reviewer,

We appreciate you for your precious time in reviewing our paper and providing valuable comments. It was your valuable and insightful comments that led to possible improvements in the current version. The authors have carefully considered the comments and tried our best to address every one of them.

We are uploading our point-by-point response to the comments, an updated manuscript with red highlighting indicating changes, and a manuscript without track changes.

Best regards,

**Reviewer 1:** *In this paper, the authors have presented the design of a compact multiple-input multiple-output antenna. The reflection coefficient of the antenna confirms the suitability of the proposed antenna for sub-6 GHz multiple-input multiple-output wireless systems. Following are the suggestions to the authors:*

***Concern # 1:*** In the abstract, the abbreviation MIMO should be defined. All the abbreviations used first time in the paper should be defined*.*

**Author response:** Agreed.

**Author action:** The abbreviation MIMO is defined in Abstract of the revised manuscript.

***Concern # 2:*** *Section numbering is missing.*

**Author response:** The Section number is missing due to the template of the PLoS One journal.

***Concern # 3:*** *In the introduction, some more recent literature related to sub-6GHz multiple-input multiple-output antennas should be included.*

**Author response:** Agreed.

**Author action:** Several recent published papers related to sub-6 GHz MIMO antenna are added to the revised manuscript as ref [9, 10, 15].

***Concern # 4:*** *At the end of the introduction section, the structure of the paper should be given.*

**Author response:** Agreed.

**Author action:** The structure of the paper is added at the end of the Introduction Section in the revised manuscript.

***Concern # 5:*** *The dimensions of the antenna should be summarized in a table.*

**Author response:** Agreed.

**Author action:** The dimensions of the antenna are summarized in Table 1 of the revised manuscript.

***Concern # 6:*** *In Table 1, the unit of the dimension should be corrected as λ^3.*

**Author response:** Agreed.

**Author action:** The unit of the dimension is corrected in the revised manuscript.

***Concern # 7:*** *Appropriate tense should be used in conclusion, please rephrase first sentence of this section.*

**Author response:** Agreed.

**Author action:** The first sentence in the Conclusion is rephrased in the revised manuscript.

***Concern # 8:*** *If not a journal requirement, please included figures within the main paper.*

**Author response:** The authors agree with the Reviewer that including figures within the main paper is better. However, according to the journal template, it is not allowed in this stage.

***Concern # 9:*** *Include some diversity parameters such as diversity gain, envelope correlation coefficient etc.*

**Author response:** Agreed.

**Author action:** The ECC and DG of the 2-port MIMO antenna have been included in the revised manuscript as Fig. 10.

***Concern # 10:*** *As mentioned above, please include some more recent papers related to the sub-6 GHz multiple-input multiple-output antennas such as ‘Compact Sub 6 GHz Dual Band Twelve-Element MIMO Antenna for 5G Metal-Rimmed Smartphone Applications’, ‘Metasurface Superstrate based MIMO Patch Antennas with Reduced Mutual Coupling for 5G Communications’, ‘A closely spaced two-port MIMO antenna with a radiation null for out-of-band suppressions for 5G Sub-6 GHz applications’ etc.*

**Author response:** The authors would like to thank the Reviewer for the valuable suggested references.

**Author action:** The suggested references are included in the revised manuscript as refs [9, 10, 15].

***Concern # 11:*** *The details of the reference [26] are incomplete.*

**Author response:** The authors would like to thank the Reviewer for pointing out the authors’ mistake.

**Author action:** The details of ref [26] are corrected in the revised manuscript.

**Reviewer 2:**

***Concern # 1:*** *The authors have presented a good research analysis, for the power divider network provide some surface current analysis.*

**Author response:** In accordance with the Reviewer comment, the surface current on the T-divider is shown in Fig. 1R. As shown, the current is equally distributed to the outputs of the divider. This makes sure that the radiating elements are excited with equal magnitude and phase.

This is the common operation of the T-divider and thus, the authors do not include this figure in the manuscript for brevity.

Fig. 1R. Simulated current distribution at 4.8 GHz on the divider.

***Concern # 2:*** *How isolation is achieved must be discussed briefly.*

**Author response:** The authors would like to thank the Reviewer for the constructive comment. The principle for high isolation of the proposed design is based on two orthogonal linear polarizations. As seen in Fig. 5, F-1 and F-3 will be excited for Port-1 operation. The coupling will occur between these feeding positions to the others, F-2 and F-4. However, the positions of F-2 and F-4 are orthogonal to F-1 and F-3. Consequently, high isolation will be obtained, as demonstrated in Section “Design a dual-polarized antenna”.

**Author action:** A brief discussion about the isolation of 2-port MIMO is added to Paragraph 1, Subsection “2-port MIMO”, Section “MIMO configurations” of the revised manuscript.

***Concern # 3:*** *Provide some information on the radiation pattern with back lobe suppression characteristics.*

**Author response:** Agreed.

**Author action:** Further discussion is added to Paragraph 3, Subsection “2-port MIMO”, Section “MIMO configurations” of the revised manuscript.

***Concern # 4:*** *Highlight some novelty of the proposed design.*

**Author response:** Agreed.

**Author action:** The novelty of the proposed design is emphasized in Paragraph 4, Section “Introduction” of the revised manuscript.

***Concern # 5:*** *For the array antenna design how did the authors reduce side lobes.*

**Author response:** For the MIMO array, the side lobes can be reduced by controlling excitation magnitudes and/or phases. This task requires complicated feeding networks as they distribute power and phase to each antenna element independently. In this paper, the authors just presented a MIMO antenna with high isolation and high gain operation, rather than proposing the feeding network.
