# Supplementary material for: A compact design of MIMO patch antenna with high gain and symmetrical radiation pattern
Source: PLoS One. 2025 Jul 10;20(7):e0325282. doi: 10.1371/journal.pone.0325282 (PMC12244613; doi:10.1371/journal.pone.0325282)
Supplement: Attachment — Submitted filename: Response letter.docx [file pone.0325282.s002.docx]

**Submission ID:** PONE-D-15830R1

**Original Article Title:** “A Compact Design of MIMO Patch Antenna with High Gain and Symmetrical Radiation Pattern”

**To:** Reviewer

**Re:** Response to reviewer

Dear Reviewer,

We appreciate you for your precious time in reviewing our paper and providing valuable comments. It was your valuable and insightful comments that led to possible improvements in the current version. The authors have carefully considered the comments and tried our best to address every one of them.

We are uploading our point-by-point response to the comments, an updated manuscript with red highlighting indicating changes, and a manuscript without track changes.

Best regards,

**Reviewer 1:**

***Concern # 1:*** Authors have addressed the comments in the revised paper. In Fig 10 of the revised manuscript, please correct ‘S-parameter’ as ‘Diversity Gain’ on the right vertical axis*.*

**Author response:** The authors would like to thank the Reviewer for pointing out our mistake.

**Author action:** Fig. 10 is corrected in the revised manuscript.

**Journal Requirements:** *Please review your reference list to ensure that it is complete and correct. If you have cited papers that have been retracted, please include the rationale for doing so in the manuscript text, or remove these references and replace them with relevant current references. Any changes to the reference list should be mentioned in the rebuttal letter that accompanies your revised manuscript. If you need to cite a retracted article, indicate the article’s retracted status in the References list and also include a citation and full reference for the retraction notice.*

**Author response:** The authors have thoroughly checked the references. All references are completed and corrected. No retracted references are cited in the paper.
